# Recent Trends in Russian Psychiatry with Particular Emphasis on Training in Women’s Mental Health

**DOI:** 10.1192/j.eurpsy.2022.168

**Published:** 2022-09-01

**Authors:** N. Ilina, N. Semenova

**Affiliations:** 1 LLC “Garnet MC”, Psychiatry, Moscow, Russian Federation; 2 V. Serbsky National Medical Center for Psychiatry and Narcology, Clinical Psychology Counseling, Moscow, Russian Federation

## Abstract

There will be two main foci to this presentation.

Firstly, Designing and implementing a new educational program entitled “Women victims of domestic violence: Detection, clinic, help” – that is mainly based on the teaching of several modules, WPA International Curriculum for Mental Healthcare Providers on Violence Against Women. Our program is a follow-on to the one held during the COVID-19 pandemic restrictions, at which interest was expressed in sharing ideas and resources.

Secondly, Informing on the recent trends in Russian perinatal psychiatry. This covers the psychiatric training in the assessment domain, case management, and service evaluation.

We will introduce and review some resources for use in women’s mental health practicals, propose innovative pedagogical structures for practical teachings, such as Problem Based Learning, ‘Vicarious Learning,’ and encourage discussion of how the practical aspects of women’s mental health teaching can be supported and enhanced.

Teaching modules and training pathways will be delivered (“not too much; not too little and in the right order”), and dimensions of quality in continuing professional development in women’s mental health (i.e., Sophistication, Credibility, Timeliness, and Utility) will be outlined.

This will be followed by a discussion exploring the different prioritization of the teaching modules across various organizations.

We urge our audience to consider it is time for psychiatric training in women’s mental health to move from the margins to the center.

